# Use of intravenous tocilizumab in pregnancy for severe coronavirus disease 2019 pneumonia: two case reports

**DOI:** 10.1186/s13256-021-03010-1

**Published:** 2021-08-07

**Authors:** Shazia Abdullah, Nihal Bashir, Nageena Mahmood

**Affiliations:** Sheikh Khalifa Medical City, Abu Dhabi, UAE

**Keywords:** Tocilizumab, COVID-19, Pregnancy, Pneumonia, Cytokine storm, Case report

## Abstract

**Background:**

Tocilizumab, an interleukin-6 inhibitor is part of many international guidelines for the management of cytokine storm associated with severe coronavirus disease 2019 with observed improvements. However, this drug is not recommended during pregnancy owing to the lack of safety data. Restriction of such medication use makes the management of coronavirus disease 2019 in pregnant women more challenging. Pregnant women are more likely to deteriorate from respiratory infections because of the immunological changes during pregnancy and the hypoxic compromise. We report the use of tocilizumab in two pregnant patients who developed severe coronavirus disease 2019 with a successful outcome. To date, there have not been any published data on tocilizumab use in pregnancy for cytokine storm syndrome associated with coronavirus disease 2019.

**Case presentations:**

In 2020, two pregnant women of Asian origin in the last trimester of pregnancy were admitted to our hospital with severe coronavirus disease 2019. Their clinical condition progressed rapidly despite maximum supportive treatments. Blood testing in the second week of illness showed rising ferritin and interleukin-6 levels, indicating the possibility of cytokine storm syndrome. Both developed respiratory failure necessitating mechanical ventilation. Due to their critical clinical condition and lack of response to supportive treatment, a decision was made to use intravenous tocilizumab therapy. Both were treated with one intravenous infusion of tocilizumab and had a successful outcome. They were extubated later and gradually weaned off supplemental oxygen. The first patient continued with her pregnancy during the hospital stay with normal fetal scans. The second patient needed an emergency cesarean section and delivered a healthy infant.

**Conclusion:**

In critical clinical situations, tocilizumab may have a role in managing coronavirus disease 2019 related cytokine storm during pregnancy.

## Introduction

The coronavirus disease 2019 (COVID-19) pandemic has affected more than 180 million people worldwide since its first identification in Wuhan, China in December 2019 [1].

The high-risk groups generally reported in the literature include patients of advanced age and those with comorbidities such as obesity, hypertension, diabetes mellitus, and chronic respiratory and cardiovascular diseases [2].

During the H1N1 influenza pandemic of 2009, pregnant women had a higher rate of hospitalization and mortality from influenza than the general population [3, 4].

Thus, as a precautionary measure for COVID-19, pregnancy had been categorized in the moderate- and high-risk groups by the UK’s Chief Medical Officer in March 2020 as well as the Centers for Disease Control and Prevention (CDC) [5, 6].

Immunological changes during pregnancy, particularly in the third trimester, make women more susceptible to severe symptoms from viral infections and hypoxic compromise, as shown in studies from the previous severe acute respiratory syndrome (SARS) and Middle East respiratory syndrome (MERS) outbreaks [7].

Certain antiviral treatments such as favipiravir and camostat, and immunomodulatory treatments such as tocilizumab and anakinra, are not routinely used for COVID-19 treatment in pregnant women, making patient management more challenging [8].

We report two cases of pregnant women admitted to our facility with COVID-19 pneumonia who deteriorated rapidly despite advanced supportive treatment and needed tocilizumab therapy during the pregnancy. To date, there is only one published case report of tocilizumab use for COVID-19 illness during pregnancy, where it was used for cardiomyopathy associated with COVID-19 [9].

## Case 1

A 29-year-old Asian lady with no previous significant past medical history presented in May 2020 to the maternity unit of our facility with a 5-day history of fever, cough, breathlessness, and diarrhea.

She was 24 weeks pregnant (gravida 4, para 3 with three previous caesarean sections). She was a nonsmoker and did not consume any alcohol. She was not taking any regular medications. On admission, she had a fever of 37.7 **°**C, blood pressure of 90/54 mmHg, and pulse of 115 beats per minute. She was found to be in mild respiratory distress with a respiratory rate of 20 breaths per minute, and oxygen saturation was 94% on room air.

**Fig. 1:**
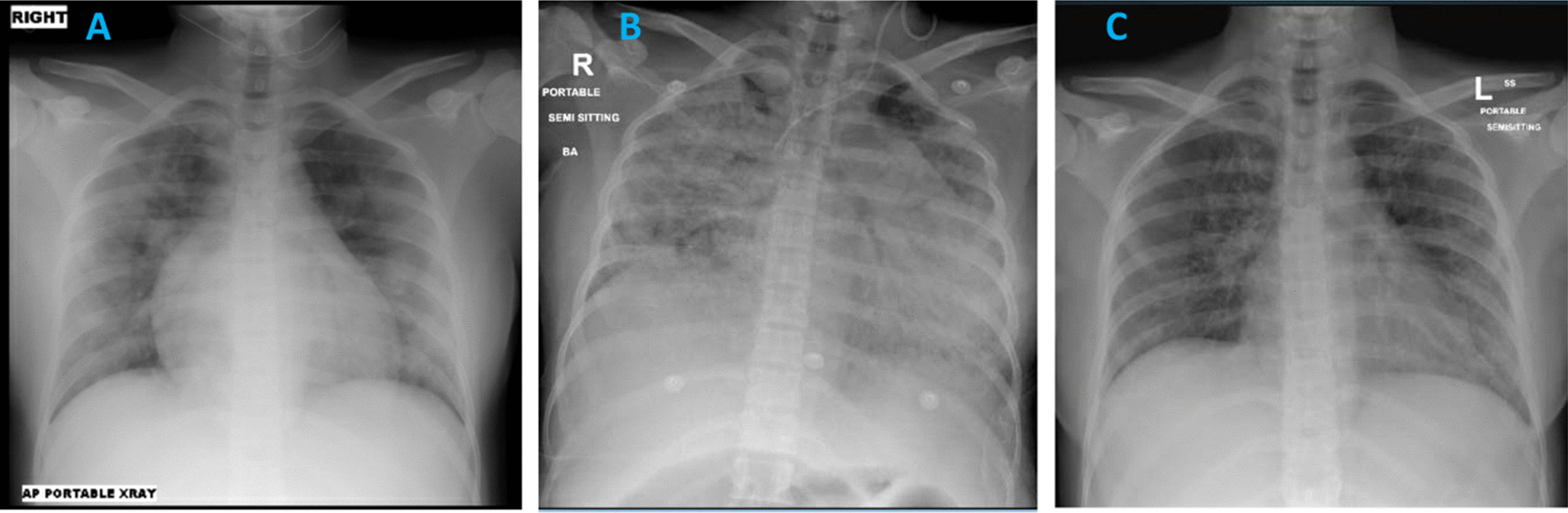
**A** Day 1 CXR showing extensive bilateral patchy pacification suggestive of COVID-19. **B** Day 5 CXR showing progression of the bilateral extensive widespread consolidation in the lungs. Left internal jugular line with the tip in the distal superior vena cava (SVC). **C** Day 28 CXR showing significant improvement in the bilateral patchy opacification

Her initial chest X-ray (CXR) (Fig. 1A) showed extensive and bilateral patchy opacification suggestive of COVID-19 illness. She tested positive for severe acute respiratory syndrome coronavirus 2 (SARS-CoV-2) on Real-time polymerase chain reaction (RT-PCR) on nasopharyngeal swab. Blood tests revealed normal renal and liver function tests, normal Hemoglobin (Hb) of 128 mg/dl, and normal platelets and white cell count including differentials. Her C-reactive protein (CRP) and interleukin-6 (IL6) levels were raised at 51 mg/L and 96 pg/ml, respectively. Her d-dimer level was 0.86 μg/ml and fibrinogen 4.2 g/L (Table1).

**Table 1:** Blood test results on admission

Blood test (normal range and units)	Case 1	Case 2
White blood cell count (4.5–11 × 10^9^/L)	5.0	9.9
Neutrophils (1.8–7.7 × 10^9^/L)	4.04	6.98
Lymphocytes (1.5–4 × 10^9^/L)	0.72	2.15
Platelet count (140–400 × 10^9^/L)	121	265
Hemoglobin (117–155 g/L)	128	115
Lactate dehydrogenase (135–214 IU/L)	424	174
Ferritin (15–150 μg/L)	282	42
D-Dimer (< 0.5 μg/ml)	0.86	1.09
Fibrinogen (2–4 g/L)	4.24	6.72
Creatinine (44–80 mmol/L)	45	31
Alanine transferase (< 41 units/L)	55	13
Aspartate transferase (< 40 units/L)	64	19
Albumin (35–52 g/L)	19	20
CRP (< 5 mg/L)	51	60
Procalcitonin (< 0.5 ng/ml)	0.26	0.06
Interleukin 6 (< 7 pg/ml)	96	28.4

CRP, C-reactive protein; g/L, grams/litre; μg/L, micrograms/litre; mg/l, milligram/litre; mmol/L, millimole/litre; ng/ml, nanogram/millilitre; pg/ml, picogram/millilitre; IU/L, international unit/litre

She was started on supplemental oxygen, hydroxychloroquine 400 mg twice daily, intravenous ceftriaxone 1000 mg twice daily, subcutaneous enoxaparin 40 mg once daily, lopinavir–ritonavir 80 mg–20 mg, two tablets twice daily, hydroxychloroquine 400 mg twice daily, and acetylcysteine 600 mg orally twice daily as per the local guidelines for COVID-19 management. Her oxygen saturation continued to drop, and after 24 hours of admission, she was on 8 L/minute of oxygen via face mask and managed in high-dependency unit.

By day 5 of admission, there was no clinical improvement. She still required respiratory support in the form of high-flow oxygen 40 L/minute and was showing signs of respiratory failure on arterial blood gases (ABGs), which revealed a pH of 7.45, pO_2_ of 55 mmHg, and pCO_2_ of 23 mmHg. Her chest X-ray (Fig. 1B) showed marked progression. In addition, she had become restless. Proning was proving difficult, and she was started on Bilevel positive airway pressure (BIPAP). Her inflammatory markers continued to deteriorate. Her IL6 was 96 pg/ml on admission and rose to 193 pg/ml. Her clinical picture was in keeping with a cytokine storm syndrome, which was not responding to her current treatment regime. A multidisciplinary meeting was called, and it was decided to administer tocilizumab 400 mg intravenously once.

Subsequently, she was intubated and ventilated because of acute respiratory failure. Her intensive care unit (ICU) admission was complicated by candidemia, treated successfully. She improved gradually and was extubated on day 16 of admission. She had an uneventful recovery with improvement in respiratory symptoms, oxygen requirements, and CXR appearances (Fig. 1C). She was discharged from the hospital after 35 days of hospitalization. Repeated fetal ultrasound scans during admission demonstrated no evidence of fetal distress. Emergency caesarean section was not indicated during hospitalization.

She later had an elective caesarean section at 38 weeks of gestation under spinal anesthesia owing to history of previous three caesarean sections and delivered a normal male child. The initial neonatal assessment was unremarkable. No further follow-up information is available.

## Case 2

A 26-year-old pregnant Asian lady at 35 weeks of gestation presented in June 2020 to the maternity unit of our hospital with 5 days of fever, dry cough, fatigue, headache, and breathlessness. She had tested positive for SARS-CoV-2 on RT-PCR on nasopharyngeal swab 5 days ago and was in self-quarantine at home. She had a background history of gestational diabetes treated with metformin 500 mg three times a day. She was a nonsmoker and did not consume any alcohol. On admission, she appeared short of breath. She had a low-grade fever of 37.5 **°**C, blood pressure of 126/78 mmHg, and pulse of 134 beats per minute. Her respiratory rate was 30 breaths per minute, and oxygen saturation was 94% on room air, improving to 99% on 4 L/minute of oxygen.

**Fig. 2:**
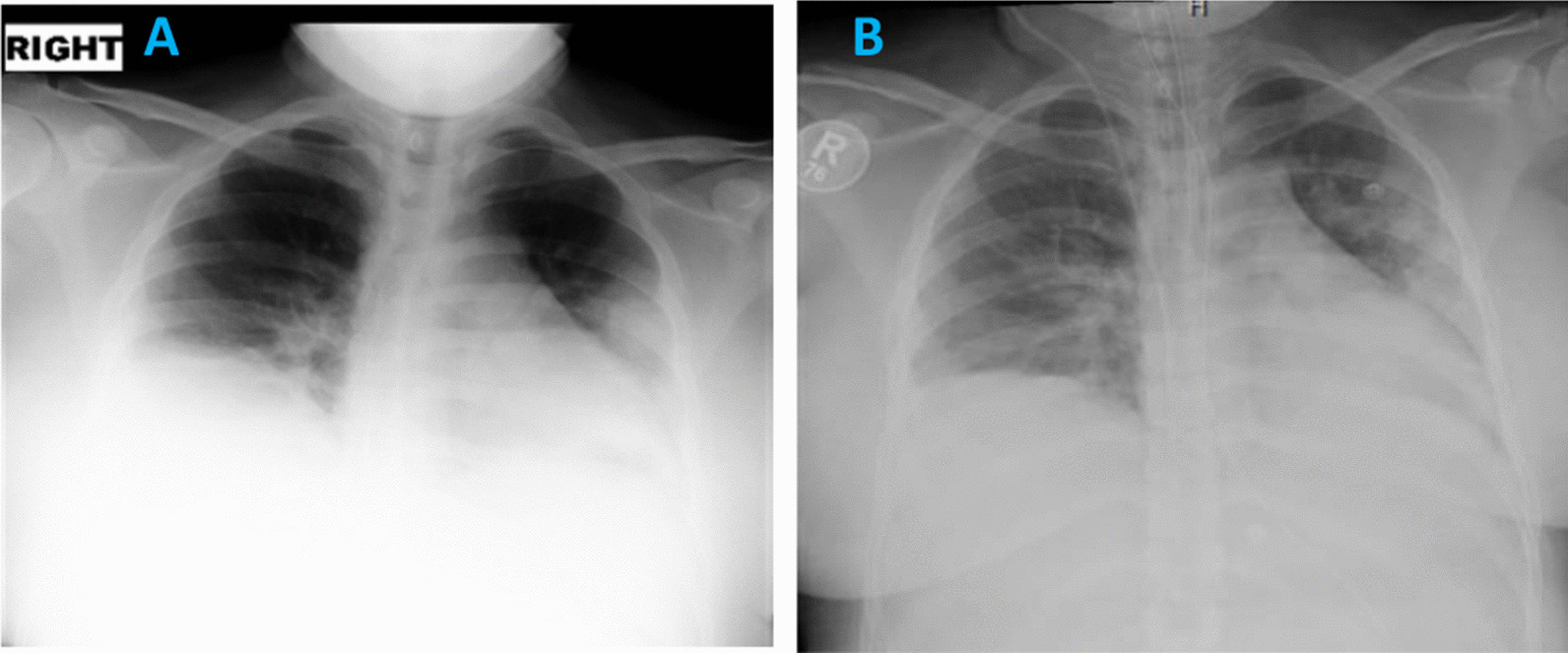
**A** Day 1 CXR showing patchy hazy confluent airspace opacity involving left mid and lower zones with blunting of the right costophrenic angle, which may represent small right-sided pleural effusion. **B** Day 2 CXR showing increasing bilateral lower zonal patchy opacification. Increased bronchovesicular and interstitial lung markings

Her CXR showed bilateral patchy infiltrates (Fig. 2A). Blood tests revealed normal renal and liver function tests, low hemoglobin of 115 mg/dl, and normal platelets and white cell count including differentials. Her CRP was raised at 60 mg/L, and IL6 levels were 28.4 pg/ml. Her d-dimer level was 1.09 μg/ml, and fibrinogen 6.72 g/L (Table 1). The fetal scan showed normal heart beats with a breech presentation.

She was started on hydroxychloroquine 400 mg twice daily orally, daily enoxaparin 40 mg subcutaneously, and oral azithromycin as per our local guidelines for management of COVID-19. The azithromycin was later changed to intravenous piperacillin–tazobactam 3000 mg intravenous four times a day. On day 2 of admission, the patient became more tachypneic with a respiratory rate of 40 breaths per minute and oxygen requirements increased to 10 L/minute via nonrebreather mask. Her chest X-ray showed progression of the bilateral changes due to COVID-19 pneumonia (Fig. 2B). She was therefore shifted to the high-dependency unit.

By day 4, the patient had deteriorated further. She was receiving oxygen of 40 L/minute through high-flow nasal cannula with a FiO_2_ 100%. Arterial blood gas testing showed a pH of 7.12, pO_2_ of 104 mmHg, and pCO_2_ of 42 mmHg. Repeat blood testing revealed a rise in ferritin and IL6 levels indicating the possibility of cytokine storm.

As the patient was deteriorating with increasing respiratory distress, a decision was made to administer intravenous tocilizumab at a dose of 600 mg. An obstetric ultrasound showed signs of fetal distress, and therefore the baby was delivered by caesarean section. The patient’s condition remained critical, and following the delivery, she was intubated and transferred to the main hospital ICU. Following delivery, she was also started on oral favipiravir 600 mg twice daily and camostat 200 mg three times a day orally as per the local guidelines.

She improved and was extubated 2 days later. Her oxygen saturation improved to 94% on 6 L/minute via nasal cannula by day 7.

Her oxygen requirements improved further over the next few days, and the supplemental oxygen was stopped in another 5 days. She was subsequently transferred back to the maternity unit and later discharged from the hospital. The initial and 6 weeks neonatal check-up was recorded normal. No further follow-up information is available.

## Discussion

We describe two cases of pregnant ladies who developed severe COVID-19 illness with evidence of acute cytokine storm. Both these patients improved clinically after use of a single dose of intravenous tocilizumab in addition to supportive treatments. Tocilizumab is generally not used during pregnancy, and to date there is only one published case report of tocilizumab use during pregnancy for severe COVID-19 illness.

During the early phase of the pandemic, clinical trial data to guide definitive treatments for COVID-19 was not available. In UAE, similar to other parts of the world, cases were approached by local and national guidelines provided by expert consensus.

A subgroup of patients with severe COVID-19 can develop an exaggerated immune response named cytokine storm syndrome that, if untreated, can lead to acute respiratory distress syndrome (ARDS) and multiple organ failure. Clinical features of this syndrome are a persistent fever, hyperferritinemia, cytopenia, and elevation of inflammatory markers such as C-reactive protein and IL6 [10].

Timely control of the cytokine storm in its early stage through immunomodulatory therapies is the key to improving the treatment success rate and reducing the mortality rate of patients with COVID-19 [11]. Tocilizumab is one such contender and is now part of many international guidelines for use in severe-to-critical COVID-19 [12].

Tocilizumab is a humanized monoclonal antibody against both the soluble and membrane-bound interleukin-6 receptor and therefore useful when IL6 is high as part of the COVID-19-associated cytokine storm. It is recommended for the treatment of severe rheumatoid arthritis, systemic juvenile idiopathic arthritis, giant cell arteritis, and life-threatening cytokine release syndrome induced by chimeric antigen receptor T-cell therapy. In China, a retrospective study on 21 patients with severe COVID-19 pneumonia treated with intravenous tocilizumab demonstrated improvement in oxygen requirements in 75% of the treated patients [13]. Another study from Italy using intravenous tocilizumab demonstrated a significantly greater survival when compared with standard therapy in patients with severe COVID-19 [14].

Tocilizumab, however, is not recommended in pregnancy. Pregnant women are deemed just as susceptible to severe COVID-19 pneumonia as their nonpregnant peers. Once critical disease occurs, the mortality rate is 49%. The only reason tocilizumab is not used in pregnancy is that there is no experience in pregnancy and, hence, safety cannot be guaranteed. Whenever clinical trials were done on tocilizumab, pregnant women were excluded. Tocilizumab is a monoclonal antibody Immunoglobulin G (IgG) that does not cross the placenta during the first trimester [15]. Thus, congenital anomalies are not associated with its use. As pregnancy advances, the transfer of tocilizumab across the placenta increases, reaching a maximum in the third trimester [16].

Reports of inadvertent use of tocilizumab indicated a slightly increased risk of preterm labor compared with the background population [17, 18].

The confounding factors are the seriousness of the disease itself and additional medication used. There are reports that most serious medical conditions have an association with preterm labor. Therefore, in the context of serious illness, there is no justification for withholding a potentially lifesaving drug in view of pregnancy. We used tocilizumab in pregnant women who developed severe COVID-19 pneumonia and critical disease with good results as witnessed in this case report. We feel strongly that pregnant women should not be denied life-saving medication based on pregnancy alone, especially when the benefit outweighs the risk.

## Conclusion

Tocilizumab may be used during pregnancy in patients who become critically unwell with cytokine storm associated with severe COVID-19 illness. The decision has to be made on a case-by-case basis by a multidisciplinary team who have experience using this drug.

## Data Availability

The data supporting the findings of this case report are available within the article and its supplementary information files.

## Abbreviations

SARS: Severe acute respiratory syndrome
MERS: Middle East respiratory syndrome
RT-PCR: Real-time polymerase chain reaction
Hb: Hemoglobin
WCC: White cell count
CRP: C-reactive protein
IL6: Interleukin 6
ABGs: Arterial blood gases
BIPAP: Bilevel positive airway pressure
IgG: Immunoglobulin G
